# Multiple infections of *Anaplasma platys* variants in Philippine dogs

**DOI:** 10.14202/vetworld.2016.1456-1460

**Published:** 2016-12-20

**Authors:** Adrian Patalinghug Ybañez, Rochelle Haidee Daclan Ybañez, Naoaki Yokoyama, Hisashi Inokuma

**Affiliations:** 1 Department of Biology and Environmental Studies, College of Sciences, University of the Philippines Cebu, Lahug, Cebu City 6000, Philippines; 2 Department of Research, Gullas College of Medicine, University of the Visayas, Banilad, Mandaue City 6014, Cebu, Philippines; 3 Department of Veterinary Clinical Science, Obihiro University of Agriculture and Veterinary Medicine, Obihiro, Inada Cho, Hokkaido 080-8555 Japan; 4 Research Unit for Molecular Diagnosis, National Research Center for Protozoan Diseases, Obihiro University of Agriculture and Veterinary Medicine, Obihiro, Inada Cho, Hokkaido 080-8555, Japan

**Keywords:** *Anaplasma platys*, dogs, Philippines, variants

## Abstract

**Aim:**

*Anaplasma platys*, the causative agent of infectious canine cyclic thrombocytopenia, is a tick-borne pathogen that also has been implicated as potentially zoonotic. To provide molecular evidence on the multiple infections of *A. platys* variants in Philippine dogs.

**Materials and Methods:**

DNA fragments of *A. platys* from infected dogs in the Philippines were molecularly characterized. For screening, 25 dogs suspected to have canine anaplasmosis were tested using a 16S rRNA-based nested polymerase chain reaction (PCR). Infection was confirmed by sequencing of positive amplicons. Second round PCR targeting a longer 16S rRNA fragment was subsequently performed on the first round PCR amplicons of the positive samples. Further characterization using the heat-shock operon (*groEL*) gene was also performed on the *A. platys*-positive samples.

**Results:**

10 16S rRNA sequences were obtained and found 99.6-100% identical to each other and 99.6-99.7% identical to the closest registered *A. platys* sequences. On the other hand, 36 *groEL* clone sequences were obtained and found to be 85.1-99.8% identical with each other and 85.0-88.9% identical to the closest previously registered *A. platys* sequences. Four dogs were found coinfected with 2-3 *groEL* variant sequences. Phylogenetic analyses suggest that the detected *A. platys* in the Philippines may represent unique variants.

**Conclusion:**

*A. platys* variants were detected in Philippine dogs. Coinfection of different *A. platys* variants in dogs was also demonstrated. The present study may indicate the potential genetic diversity of *A. platys* in the country.

## Introduction

*Anaplasma platys* is a Gram-negative intracellular bacteria of the family Anaplasmataceae [1] that has recently been reported to infect humans [2-4]. In the Philippines, it has been detected in ticks [5] and dogs [6]. Nearby countries including Thailand [7], China [8], Korea [9], Japan [10], and Taiwan [11] already have reports of detection and characterization of this pathogen.

Characterizations of *A. platys* based on the heat-shock operon gene (*groEL*) are limited. This particular gene has been used to identify genotypes of a related *Anaplasma* species. Pathogenicity of *Anaplasma* sp. maybe related to its genotype [12], although detailed investigations using *groEL* in assessing clinical signs or effects to the animal has been limited.

Only few reports on *A. platys* detection in the Philippines are available. The present study aimed to detect and molecularly characterize *A. platys* based on the 16S rRNA and *groEL* genes from infected dogs in the country. The obtained sequences were further subjected to phylogenetic analyses using different methods.

## Materials and Methods

### Ethical approval

The study was performed in accordance with the Institutional Animal Care and Use Committee guidelines of Southwestern University, Cebu and the Animal Welfare Act of the Philippines (RA8485), and with the approval of the attending veterinarians and proprietor of the veterinary establishment.

### DNA sample from dogs

DNA samples were obtained from blood of 25 dogs that were presented at the GPY Veterinare Animale Group of Veterinary Clinics, Cebu, Philippines. These dogs were thrombocytopenic and exhibited clinical signs suggestive of canine anaplasmosis [13] and were found serologically negative for *Ehrlichia canis* using an Immunocomb^®^ (Biogal, Israel) commercial test kit [14]. DNA was extracted using procedures as previously described [5].

### 16S rRNA polymerase chain reaction (PCR) assay

For the 16S rRNA PCR, DNA samples were tested using a previously described method [5] with slight modification to confirm infection. Briefly, outer primer pair fD1/Rp2 and inner primer pair EHR16SD/EHR16SR (for screening) were used to amplify a final 345-bp-target of *Ehrlichia/Anaplasma* spp. Those found positive with the EHR16SD/EHR16SR screening PCR were further analyzed. Using the first round fD1/Rp2 amplicons, hemi-nested PCRs were performed utilizing the primer pairs fD1/EHR16SR and EHR16SD/Rp2 targeting a total of approximately 1400-1600 bp. A final volume of 10 and 25 µl was set for the first and final round PCRs using a similar method, respectively [15]. The negative and positive controls used were double distilled water and *A. bovis* [16], respectively.

### groEL-based PCR assay

DNA samples which were found positive during the screening PCR were further analyzed using a *groEL-*based nested PCR assay. In the first round, primers EEgro1F and EEgro2R [17] were used employing procedures as previously described [18]. For the second round PCR, newly designed primers, i.e. APl136F (CCRGAGATKACKAAGGATGGC) and APl590R (CCRCGRTCAAAY TGCATACC) that targeted a 455 bp fragment were used. Similar final PCR volumes were used [18]. Step-down PCR method (with 2°C increment) was employed in the second round PCR with a starting and final annealing of 62°C and 52°C, respectively.

### Cloning, sequencing, and sequence comparison

Final amplicons were purified using a Gel Extraction Kit (QIAGEN, Valencia, CA, USA) and were cloned using a TA cloning kit (Invitrogen, USA). DNA sequencing was performed as described previously [18]. Obtained 16S rRNA and *groEL* sequences were aligned as previously described [18] to obtain partial but longer DNA fragments. Sequence comparison was also performed as previously described [18]. All sequences obtained in this study were registered at GenBank, USA.

### Phylogenetic analyses

All sequences were manually trimmed to include only the sequence of interest. Percent identities were computed, and multiple sequence alignments were performed as previously described [18]. Analyses by neighbor-joining using maximum composite likelihood, by maximum likelihood with prior best model testing using MEGA 5 [19], and by Bayesian inference using MrBayes 3.2 [20] guided by the prior best model test results from MEGA 5 were employed. A total of 1000 bootstrap replications and 1,000,000 generations were used in the analyses using MEGA 5 and MrBayes 3.2, respectively. Tree results from MrBayes 3.2 were viewed using the FigTree v1.3.1 (http://tree.bio.ed.ac.uk/software/figtree/).

## Results and Discussion

The screening PCR revealed 10 positive dogs. Obtained sequences (KP006398-KP006406) after alignment (1467 bp) were 99.6-100% identical to each other and 99.6-99.7% identical to the closest registered *A. platys* sequences from the Philippines and Thailand. From the *A. platys*-positive samples, further characterization using the *groEL* resulted to 36 clone sequences (KP027333-KP027368), which were 85.1-99.8% identical with each other and 85.0-88.9% identical to the closest registered *A. platys* sequences.

From the positive dogs, four were found coinfected with 2-3 *groEL* variant sequences. To the best of our knowledge, several studies have reported multiple infections of different tick-borne pathogens in dogs [2,3,7,21], but multiple infections with different variants of a pathogen in an animal have not been reported until this study. While coinfections can produce more severe signs [7], further studies are needed to determine if coinfections with different variants of *A. platys* in dog have an effect on the clinical expression of the disease.

**Figure-1:**
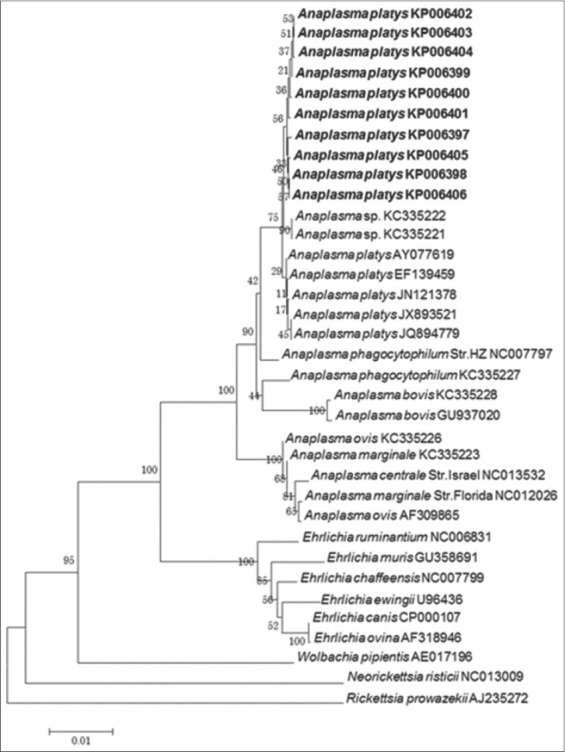
Phylogenetic analysis of *Anaplasma platys* detected from Philippine dogs based on the 16S rRNA gene using maximum likelihood method. *Rickettsia prowazekii* was set as the out-group. Sequences obtained in the study are set in bold.

Regardless of the phylogenetic methods used, analyses based on the 16S rRNA gene showed that the obtained sequences formed a different *A. platys* subclade supported by moderate to high bootstrap values (Figure-1) or posterior probabilities (figure not shown). Interestingly, neutrophil-tropic *Anaplasma* spp. isolates detected from Mediterranean ruminants [22] clustered with the Philippine variants. Due to low intra-species variability of the 16S rRNA gene, analyses based on other genes such as *groEL* are needed to validate potential phylogenetic relationships.

**Figure-2:**
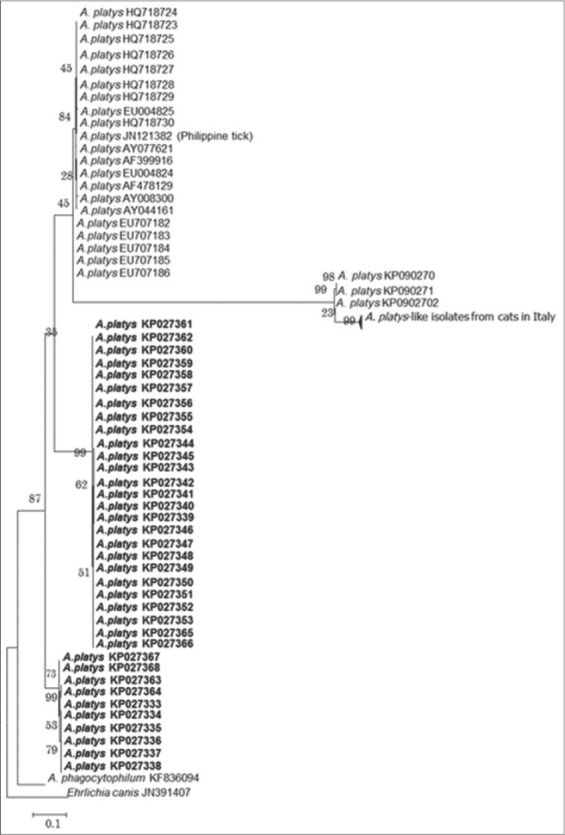
Phylogenetic analysis of *Anaplasma platys* detected from Philippine dogs based on the heat-shock operon (*groESL*) gene using maximum likelihood method (utilizing nucleotide sequences). *Ehrlichia canis* was set as the out-group. Sequences obtained in the study are set in bold.

Based on the *groEL* gene, phylogenetic analyses revealed that the detected Philippine *A. platys* variants formed a unique cluster (Figure-2). Owing to the higher number of newly obtained sequences in this study and higher variability of the gene, more subclades within the *A. platys* group were seen in the latter analyses. Moreover, new *A. platys* and *A. platys*-like isolates from Italy [23] were found to be in a unique subclade that appears to cluster differently from the detected Philippine *A. platys* variants. In this study, *groEL* was used to show the different variants of *A. platys* because of its higher intra- and inter-species variation than the 16S rRNA [13,18]. Owing to this advantage, the *groEL* is used for genotyping other species [12,24]. Moreover, phylogenetic results using this gene have been shown to produce similar results using the *ankA*, *gltA*, and 16S rRNA [1,25-28].

Identities of the newly obtained sequences to registered *A. platys* sequences were confirmed by high similarities in two genes (16S rRNA and *groEL*). Phylogenetic analyses’ results suggest that the detected *A. platys* in the Philippines may represent unique variants as the obtained sequences branched separately under the *A. platys* clade. These results can indicate the potential genetic diversity of *A. platys* in the country. This study supports the usefulness of *groEL* in investigating genetic diversities in *Anaplasma* species. On the other hand, further studies on the multiple infection of several *A. platys* variants in dogs that may also be coinfected with *E. canis* [29,30], which has a common vector and is present in the Philippines.

## Conclusion

*A. platys* variants were detected in Philippine dogs. Coinfection of different *A. platys* variants in dogs was also demonstrated. The present study may indicate the potential genetic diversity of *A. platys* in the country.

## Authors’ Contributions

APY and RHDY conceptualized the study and analyzed and wrote the manuscript. RRV contributed in the data analysis. NY and HI gave valuable insights and support in the conduct of the study.
